# Ubiquitin Conjugation of Hepatitis B Virus Core Antigen DNA Vaccine Leads to Enhanced Cell-Mediated Immune Response in BALB/c Mice

**DOI:** 10.5812/kowsar.1735143X.689

**Published:** 2011-08-01

**Authors:** Jian-Hua Chen, Yong-Sheng Yu, Hong-Hong Liu, Xiao-Hua Chen, Min Xi, Guo-Qing Zang, Zheng-Hao Tang

**Affiliations:** 1 Department of Infectious Diseases, Sixth People’s Hospital Affiliated to Shanghai Jiaotong University, Shanghai, China

**Keywords:** DNA vaccine, Ubiquitin, Hepatitis B core antigen

## Abstract

**Background:**

Nearly 350 million persons worldwide are chronically infected with hepatitis B virus (HBV). Ubiquitin (Ub) is a highly conserved small regulatory protein, ubiquitous in eukaryotes, that usually serves as a signal for the target protein that is recognised and degraded in proteasomes . The Ub-mediated processing of antigens is rapid and efficient and stimulates cell-mediated immune responses. Accordingly, Ub-mediated processing of antigens has been widely used in chronic-infection and cancer studies to improve immune response.

**Objectives:**

Many clinical trials have shown that DNA vaccine potency needs to be greatly enhanced. Here, we report a new strategy for designing an HBV DNA vaccine using the ubiquitin (Ub) sequence. The aim of this study was to investigate a novel DNA vaccination, based on the expression of HBV core antigen (HBcAg), fused to Ub to enhance DNA vaccine potency.

**Materials and Methods:**

Mouse ubiquitin fused to the HBcAg gene and cloned into the eukaryotic vector pcDNA3.1 (-). BALB/c mice were immunized with recombinant pUb-HBcAg or pHBcAg DNA vaccine. Lymphocyte proliferation assay, intracellular IFN-γ assay, CTL cytotoxicity assay, and antibody assay were performed to analyze the cellular and humoral immune responses to our DNA constructs.

**Results:**

HBcAg was expressed effectively in the COS-7 cells that were transiently transfected with pUb-HBcAg. Strong anti-HBc IgG responses were elicited in mice that were immunized with pUb-HBcAg. The endpoint titers of anti-HBc peaked at 1:656100 on the 42nd day after the third immunization. pUb-HBcAg stimulated greater lymphocyte proliferation and induced higher levels of IL-2 and IFN-γ and a greater percentage of HBcAg-specific CD8+ T cells in mice than pHBcAg. In the CTL assay, the specific lysis rate reached 56.5% at an effector:target ratio of 50:1 in mice that were immunized with pUb-HBcAg.

**Conclusions:**

pUb-HBcAg elicits specific anti-HBc responses and induces HBc-specific CTL responses in immunized BALB/c mice. Our results imply that Ub can be used as a molecular adjuvant that enhances the potency of DNA vaccines.

## 1. Background

An estimated 350 million persons worldwide are chronically infected with hepatitis B virus (HBV). HBV infection is a major global public health problem. Approximately 600,000 deaths each year are attributed to acute or chronic HBV infection [1]. Although some antiviral drugs are extremely well tolerated and suppress HBV replication effectively, they rarely eliminate intranuclear viral covalently closed circular DNA [2]. Therefore, it is necessary to develop an alternative, effective therapeutic approach for chronically infected patients. The antigen-encoding DNA vaccine, which can effectively induce humoral and cellular immune responses, has become an attractive immunization strategy against a variety of pathogens, including HBV [3][4][5]. A prophylactic vaccine that based on hepatitis B surface antigen is an effective way of reducing the global incidence of hepatitis [6], but it does not work therapeutically [7]. HBV core antigen (HBcAg) possesses unique immunological features. Patients who successfully clear the virus usually have efficient HBcAg-specific cytotoxic T lymphocyte (CTL) responses [8][9]. Plasmid DNA that encodes HBcAg elicits humoral and cellular responses in many animal models [5][10][11]. Therapeutic DNA vaccination is a promising strategy for controlling chronic infections. However, this approach has not been as successful as initially anticipated for chronic hepatitis B. The application of DNA vaccines in humans has been limited due to their low immunogenicity [12]. Many attempts have been made to enhance the potency of DNA vaccines, including codelivery of a cytokine [13] and insertion of certain sequences that enhance immune responses, such as cytokine and chemokine genes, into the vector [14][15].

It is generally accepted that the primary cause of viral persistence during HBV infection is an inadequate antiviral response to viral antigens. Individuals who are chronically infected with HBV generally have low to undetectable CTL responses to HBV antigens. Specific CD8+ T cells function as CTLs, eliminating HBV [16][17][18]. Antigen presentation to CD8+ T cells is mediated by MHC class I molecules, expressed on the surface of antigen-presenting cells. Prior to such presentation, antigens must be ubiquitinated and processed into suitable antigenic peptides by the ubiquitin-proteasome system (UPS) [19][20][21]. The UPS is a highly selective ATP-dependent proteolytic system in all eukaryotic cells that underlies antigen presentation. Ubiquitin (Ub), a highly conserved, 76-amino-acid polypeptide that is expressed in all eukaryotes, is a part of the UPS. The attachment of ubiquitin to a protein is the initial signal for its targeted degradation. When a protein is fused to ubiquitin, its degradation by the proteasome and presentation can be rapided, resulting in effectively induced immune responses. This strategy has been applied to DNA vaccines to improve immune responses by enhancing the production of antigenic peptides that are presented by MHC class I molecules [20][21][22].

## 2. Objectives

In the study, we constructed expression plasmids encoding mutant Ub-fused HBcAg and demonstrated that this fusion DNA vaccine induces humoral and cellular immune responses against HBV.

## 3. Materials And Methods

### 3.1. Cell Line and Plasmids

COS-7 cells were maintained in Dulbecco's modified Eagle's medium (Invitrogen, Gaithersburg, MD, USA), supplemented with 10% fetal bovine serum (Gibco, USA), 100 U/ml penicillin, and 100 μg/ml streptomycin. The H-2(d) mastocytoma cell line P815/c (expressing HBV c antigen) was preserved in our lab. The pADR plasmid, containing full-length HBV DNA, was provided by Prof. Yuan Wang, Shanghai Institute of Biochemistry and Cell Biology, Chinese Academy of Sciences. The eukaryotic vector pcDNA3.1 (-) was provided by Prof. Huaidong Song, Ruijin Hospital, Shanghai Jiao Tong University School of Medicine.

### 3. 2. Plasmid Construction and Preparation

We used pcDNA3.1 (-) as our expression vector in eukaryotic cells to construct two plasmids: pcDNA3.1 (-) -Ub-HBcAg and pcDNA3.1 (-) -HBcAg. To construct the Ub-HBcAg fusion plasmid, the gene encoding mutant ubiquitin, whose C-terminal Gly residue was replaced with Ala, was amplified by PCR from genomic DNA from BALB/c mouse liver using primers A and B [23]. The HBcAg gene, whose N-terminal Met residue was replaced with Arg according to the N-end rule, was amplified from the pADR plasmid by PCR using primers C and D. The primers are shown in Table Ub-HBcAg was spliced by overlap extension by PCR with primers A and D. The purified Ub-HBcAg fragment was inserted into EcoRI/HindIII-digested pcDNA3.1 (-).

**Table:** Primers of Ubiquitin and HBcAg

**Gene Name**	**Nucleotide Sequence (5'→3' )**	**RestrictionEnzyme Site**
Ub	A: CGCA**GAATTC^a^**ATGCAGATCTTCGTGAAG	EcoR I
	B: ATTCTTTATACGGGTCAATGT**CTCT^b^AGC^c^**ACCTCTCAGGCGAAGGACCAGG	
HBcAg	C: CCTGGTCCTTCGCCTGAGAGGT**GCT^c^AGA^b^**GACATTGACCCGTATAAAGAAT	Hind III
	D: GCG**AAGCTT^a^**CTAACATTGAGATTCCCGAG	
HBcAg d	E: GATA**GGATCC^a^**ATGGACATTGACCCGTAT	BamH I
	F: GCG**AAGCTTC^a^**TAACATTGAGATTCCCGAG	Hind II

^a^ Restriction enzyme site

^b^ The Ub C-terminal glycine had been replaced with alanine

^c^ HBcAg with a modified N-terminal methionine residue was replaced by arginine

^d^ For the construction of pHBcAg plasmid, the HBcAg gene was PCR-amplified with primers

To construct the HBcAg DNA vaccine, the HBcAg gene was PCR-amplified from pADR using primer E with a BamH I site and the antisense primer F with a Hind III site. The PCR product was digested with BamHI/Hind III and ligated into pcDNA3.1 (-). All plasmids were confirmed by restriction enzyme digestion and sequence analysis. Plasmids were grown in Escherichia coli DH5α and purified using an endotoxin-free purification kit (Qiagen, Germany). The purified plasmids were resuspended in sterile PBS to 500 μg/ml.

### 3. 3. In Vitro Transfection, Western Blotting

For transfection, COS-7 cells (5 × 106 cells/ml) were used to seed 6-well plates in DMEM supplemented with 10% FBS and incubated for 24 h at 37°C in a humidified incubator, 5% CO2. Cells were transiently transfected with pUb-HBcAg, pHBcAg, or pcDNA3.1 (-) using Lipofectamine 2000 (Invitrogen, USA) per the manufacturer. Forty-eight hours after transfection, cells were harvested to analyze HBcAg by western blot, as described elsewhere [24]. Mouse monoclonal anti-human HBcAg (Santa Cruz, USA) was used as the primary antibody, and horseradish peroxidase-conjugated goat anti-mouse IgG was used as the secondary antibody.

### 3.4. RT-PCR

In order to detect expression of the DNA vaccines in our eukaryotic system, we extracted total RNA from transfected cells and analyzed it by RT-PCR. For each PCR reaction, 2 μl of cDNA product of the reverse-transcription reaction was used as the template. The PCR primers were as follows: Ub-HBcAg forward primer: 5′-CGCAGAATTCATGCAGATCTTCGTGAAG-3′ and reverse primer: 5′-GCGAAGCTTCTAACATTGAGATTCCCGAG-3′; HBcAg forward primer: 5′GATAGGATCCATGGACATTGACCCGTAT-3′ and reverse primer: 5′-GCGAAG CTTCTAACATTGAGATTCCCGAG-3′, and the housekeeping gene β-actin, forward primer: 5′-CATCTCTTGCTCGAACA-3′ and reverse primer: 5′-ATCATGTTTGAG ACCTTCAACA-3′. The PCR comprised the following steps: 94°C for 10 min and 32 cycles of 94°C for 60s, 54°C for 45s, and 72°C for 120s. Finally, the reactions were incubated at 72°C for 10 min. The PCR products were examined by agarose gel electrophoresis.

### 3. 5. Mice and DNA Vaccination

Female BALB/c mice (H-2(d)), 6-8 weeks old, were acquired from Shanghai Experimental Animal Center of Chinese Academy of Sciences and kept under pathogen-free conditions. The animal experiments were approved by the institutional ethical committee of the Sixth People's Hospital, Shanghai Jiaotong University. Mice were divided into 5 groups, with 8 mice in each group. To increase the efficiency of the gene transfer, the tibialis anterior muscle was injected with a total of 100 μl of 0.5% aethocaine per mouse. After 24 h, 100 μg pUb-HBcAg, 50 μg pUb-HBcAg, 100 μg pHBcAg, 50 μg pHBcAg, or 100 μg pcDNA3.1(-) was injected into the same muscle in 200 μl of PBS. The mice were immunized intramuscularly on Days 0, 14, and 28.

### 3. 6. Detection of HBcAg-Specific IgG

Blood was collected on Days 0, 14, 28, and 42 after primary immunization from anesthetized mice by retro-orbital bleeding. Serum anti-HBc was measured by enzyme-linked immunosorbent assay (ELISA) (Diagnostic Reagent Center of Shanghai Municipal Infectious Diseases Hospital, Shanghai, China) in 96-well microtiter plates coated with HBcAg. Serum was serially diluted in PBS with 5% nonfat milk (starting from 1:100) and incubated in microtiter plates for 1 hour at 37°C. Plates were washed, and horseradish peroxidase-conjugated goat anti-mouse IgG (1:1000) was added to the wells and incubated at room temperature for 1 hour. After extensive washing, the plates were incubated at room temperature for 30 min with 100 μl of substrate in the presence of hydrogen peroxide. The absorbance at 450 nm was measured. Endpoint titers were defined as the highest serum dilution that resulted in an absorbance value 2 times greater than that of negative control sera.

### 3.7. Lymphocyte Preparation and Lymphocyte Proliferation Assay

Mice were anesthetized and sacrificed 2 weeks after the last immunization, and their spleens were homogenized over 200 gauge nylon mesh. Splenocytes were collected, treated with lysis buffer to eliminate red cells, washed, and resuspended in RPMI-1640 with 10% FBS. Lymphocytes were derived from splenocytes using nylon wool columns. Single-cell suspensions of lymphocytes (5 × 105 cells/well) were grown in 96-well plates, stimulated in vitro with 10 μg/mL MBP-HBcAg (purified in our laboratory) [25], and incubated at 37°C in 5% CO2 for 72 h. Cultures were incubated with 10 μL CCK-8 solution (Beyotime Institute of biotechnology, Haimen, China) for 4 h at 37ºC. The absorbance was read at 450 nm.

### 3.8. Measurement of Cytokine Production In Vitro

To determine whether T helper cells were activated, we measured cytokines (IL-2, IFN-γ, IL-4, and IL-10) that reflected the Th1 and Th2 responses. Splenocytes from mice in each group were cultured in 96-well culture plates in the presence of 10 μg/mL MBP-HBcAg for 4 days, and the supernatants were harvested. Cytokines were measured using commercial ELISA kits, per the manufacturer's protocol (R&D Systems, Minneapolis, MN, USA). Data are expressed as pg/ml.

### 3.9. Measurement of Intracellular Cytokines in Proliferating T Cells

IFN-γ production was measured by intracellular staining and flow cytometry. Spleen cells from immunized mice were plated in 96-well plates and stimulated with MBP-HBcAg (final concentration 10 μg/mL) for 4 days at 37°C in a humidified atmosphere, 5% CO2. Proliferating T cells were suspended in complete RPMI 1640 and stimulated for 6 h in the presence of 25 μg/mL phorbol 12-myristate 13-acetate, 1 μg/mL ionomycin, and 1.7 μg/mL monensin (Sigma, USA). After being washed with PBS, the cells were stained with FITC-conjugated anti-CD8°C mAb (eBioscience, USA) for 30 minutes at 4°C, washed with PBS, fixed with 4% paraformaldehyde, and permeabilized with PBS containing 0.5% saponin (both from BD Shanghai, China). Cells were incubated with PE-labeled anti-interferon-γ (IFN-γ) (eBioscience, USA) for 30 minutes at 4°C, washed with PBS, and analyzed by flow cytometry.

### 3.10. HBV-Specific CTL Activity

Mouse splenocytes from immunized mice were stimulated in vitro for 5 days with 10 μg/ml MBP-HBcAg and 20 IU/ml recombinant murine IL-2. The restimulated splenocytes (5 × 106/mL) were used as effectors. The P815/c cell line was used as the target cell. P815/c cells were seeded at 5 × 104 cells/well in 96-well plates and cultured for 12 h. Effector cells were incubated with P815/c at various effector/target (E/T) ratios (12.5:1, 25:1, or 50:1) for 4 h. The HBcAg-specific CTL activity was measured by lactate dehydrogenase (LDH) release assay per the manufacturer's instructions using the CytoTox 96® Non-Radioactive Cytotoxicity Assay (Promega, USA). The absorbance values from supernatants were recorded at OD 450 nm. The percentage of cytotoxicity was calculated as follows: [(Experimental release - Effector spontaneous release - Target spontaneous release)/(Target maximum release - Target spontaneous release)] × 100%.

### 3.11. Statistical Analysis

Results were expressed as mean ± SD. The statistical significance of differences between 2 groups was determined by student's t-test, and differences between 2 or more groups were analyzed by one-factor analysis of variance (ANOVA). Data were considered statistically significant at P < 0.05.

## 4. Results

### 4. 1. Construction and Expression of DNA Vaccines

**Figure 1:**
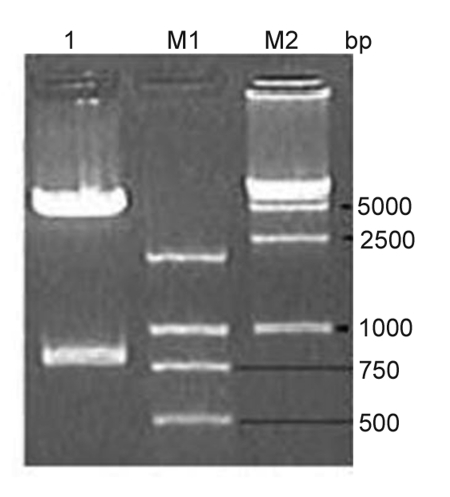
Electrophoresis of pUb-HBcAg Digested by EcoR I and Hind III. Lane 1: pUb-HBcAg Digested by EcoR I and Hind III; Lane M1: DNA Marker 2000; Lane M2: DNA Marker 15000.

**Figure 2:**
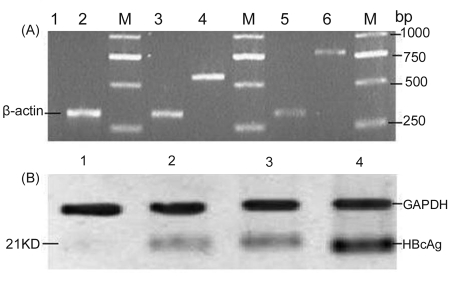
Expression of the Plasmids. (A) Gene expression of HBcAg and Ub-HBcAg, Lane M: DNA marker 2000; Lane 1: blank plasmid pcDNA3.1 (-) transfecting COS-7 cells; Lane 2,3,5: β-actin;Lane 4: plasmid pHBcAg transfecting COS-7 cells; Lane 6: plasmid pUb-HBcAg transfecting COS-7 cells. (B) Protein expression of HBcAg (about 21 kDa), Lane 1: COS-7 cells transfected with pcDNA3.1 (-); Lane 2 and Lane 3 are the same sample: COS-7 cells transfected with pUb-HbcAg; Lane 4: COS-7 cells transfected with pHBcAg

The ubiquitin gene was PCR-amplified from genomic DNA from the liver of BALB/c mice, producing a band of 552 bp; splicing of the ubiquitin and HBcAg genes by overlap extension generated a product of 780 bp. The fragment was purified and cloned into pcDNA3.1 (-). Recombinant plasmids were confirmed by restriction enzyme digestion (Figure 1) and sequencing. The sequences of the DNA vaccines were confirmed by sequencing. The expression of HBcAg or the fusion protein (Ub-HBcAg) was detected by RT-PCR and western blot 48 h after the plasmids were transfected into COS-7 cells. By RT-PCR of pHBcAg transfectants, we amplified about a product of approximately 550 bp, consistent with the HBcAg gene (552 bp), and a 780-bp product from cells that were transfected with pUb-HBcAg (Figure 2A). No similarly sized bands were seen in the negative control RNA. By western blot, 21-kD proteins were expressed in the lysates of COS-7 cells that were transfected with pUb-HbcAg and pHBcAg. pcDNA3.1 (-) transfectants did not express any such proteins. (Figure 2B). These results confirm that HBcAg and Ub-HBcAg can be expressed in a eukaryotic system.

### 4. 2. Production of HBc-Apecific Antibody by DNA Vaccination

**Figure 3:**
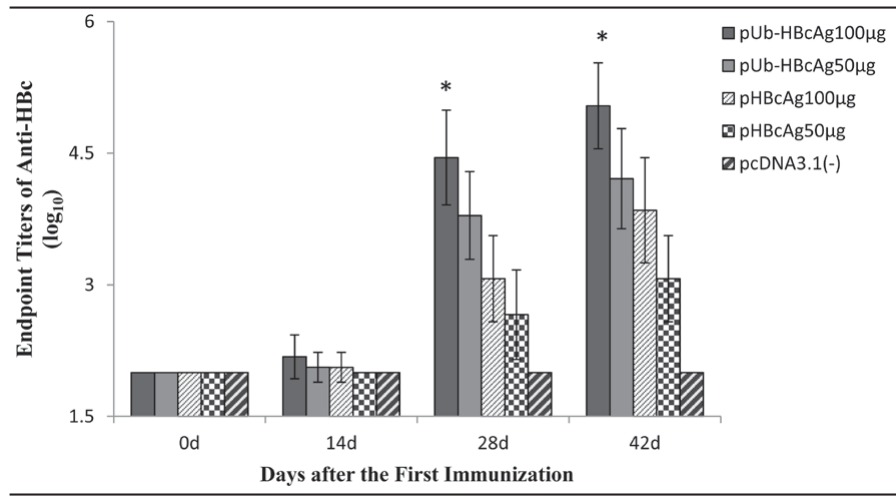
HBcAg-Specific IgG Titer in the Sera of Mice Immunized with Various Formulations. The initial dilution of each serum from immunized mouse was 1:100 and followed with serial of three-fold dilution for anti-HBc IgG detection. Data is from one representative experiment of three performed and is presented as the geometric mean titer ± SD (n = 8). **P < 0.01 pUb-HBcAg 100μg group.

The anti-HBc response that was induced by the vaccines was evaluated by ELISA. At 14 days after priming, there was no significant difference in the endpoint titers of anti-HBc between groups. As shown in Figure 3, robust anti-HBc IgG responses were elicited in mice that were immunized with 100 μg pUb-HBcAg; the endpoint titers of anti-HBc was 1:72,900, 28 d after the first immunization. The antibody titers at 28 days were significantly higher in the group that was immunized with 100 μg pUb-HBcAg versus 100 μg pHBcAg (P = 0.001). At 42 days after priming, 100 μg pUb-HBcAg significantly enhanced antibody responses compared with pHBcAg at the same dose (P < 0.01). The endpoint titers of anti-HBc in mice that were immunized with pUb-HBcAg 100 μg peaked at 1:656,100 on Day 42. No anti-HBcAg was detected in the serum of control mice that were injected with pcDNA3.1 (-). These results indicate that pUb-HBcAg enhances humoral immune responses.

### 4.3. Lymphocyte Proliferation and Cytokine Production by Splenocytes from Vaccinated Mice

**Figure 4:**
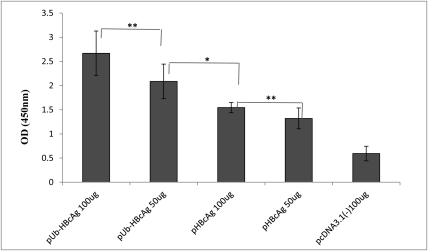
Detection of T Lymphocyte Proliferation Response. Data shown represent the mean and SD. **P < 0.05, *P <0.01.

**Figure 5:**
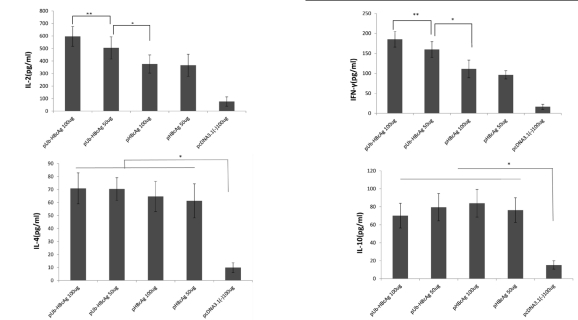
Cytokines Production in the Supernatant of Splenocytes Harvested from Immunized Mice after in Vitro Re-stimulation. Data Represent the Means ± SD (n = 8).

After 72 h, lymphocyte proliferation was measured by CCK-8 assay. Compared with the 100 μg pHBcAg and 50 μg pHBcAg groups, pUb-HBcAg induced significantly greater lymphocyte proliferation (P < 0.01). pHBcAg (100 μg) effected greater lymphocyte proliferation than 50 μg pHBcAg (P < 0.05). As seen in Figure 4, the pUb-HBcAg 100 μg group experienced the greatest lymphocyte proliferation, indicating that vaccination with Ub-fused HBcAg induces the proliferation of specific lymphocytes. Th1 (IFN-γ, IL-2) and Th2 cytokines (IL-4, IL-10) were measured in the supernatants of splenocytes ELISA. pUb-HBcAg 100 μg and pUb-HBcAg 50 μg induced higher levels of IL-2 than pHBcAg. As shown in Figure 5, IFN-γ levels in the pUb-HBcAg 100 μg group were also much higher compared with the other plasmids. Compared with pUb-HBcAg 50 μg (159.8 ± 19.55 pg/mL), pUb-HBcAg 100 μg elicited significantly higher production of IFN-γ (185.4 ± 19.63 pg/mL) (P < 0.05). Mice that were immunized with pUb-HBcAg and pHBcAg produced more Th2 cytokines (IL-4, IL-10) than the control group (P < 0.01). However, Th2 cytokine levels did not differ significantly between the pUb-HBcAg and pHBcAg groups.

### 4. 4. Specific CD8+ T Cells Response

**Figure 6:**
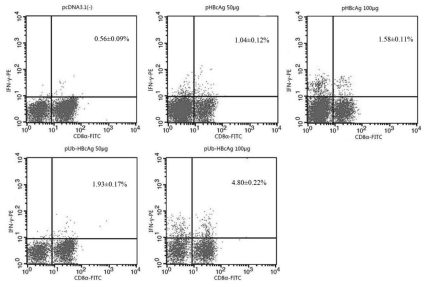
Intracellular Cytokine Expression in Spleen Cells. The whole cell population was doubly stained with fluorescent material labeled using FITC-CD8α and PE-IFN-γ antibodies. The doubly stained cells were counted and analyzed by flow cytometry. The data are the mean ± SD from eight mice per group.

Mice from each group were sacrificed, and splenocytes were isolated after the last immunization and restimulated in vitro. Cells were doubly stained with FITC-CD8α and PE-IFN-γ antibodies, counted, and analyzed by flow cytometry. IFN-γ expression was higher with pUb-HBcAg than in the pHBcAg samples. The percentage of IFN-γ-positive T cells in the CTL population was significantly higher with 100 μg pUb-HBcAg versus pHBcAg. (Figure 6). Thus, pUb-HBcAg was effective in generating CTLs.

### 4.5. HBcAg-Specific CTL Response

To evaluate the specific cytotoxicity of T lymphocytes in the various groups of immunized mice, the LDH relaxation index was measured. Splenocytes from immunized mice were restimulated in vitro with MBP-HBcAg and rmIL-2. HBcAg-specific CTL activity at various effector/target ratios is shown in (Figure 7). T lymphocytes from mice that were vaccinated with pUb-HBcAg 100 μg killed 52.0 ± 4.5% of target cells at an effector: target ratio of 50:1, significantly higher than with pUb-HBcAg 50 μg (42.0 ± 4.8%) (P < 0.01). pUb-HBcAg 50 μg elicited a greater CTL response, compared with pHBcAg 100 μg, at an effector:target ratio of 50:1 (P < 0.05). At 25:1, the CTL response against pUb-HBcAg 100 μg was higher versus the other groups (P < 0.05). These results indicate that mice that are immunized with pUb-HBcAg develop strong, specific CTL responses.

## 5. Discussion

Immune responses play a significant role in the clearance of hepatitis B virus. Protective immunity against HBV depends on both the humoral and cellular immune responses. Vigorous polyclonal and multispecific immune responses to HBV have been detected in acute infections. In contrast, defective CTL- and Th cell responses have been observed in patients who are chronically infected HBV [16]. It has been reported that specific cellular immunity against HBV is a key factor in the control of HBV infection [26][27]. One promising method is the use of DNA vaccines, which have the ability to induce antigen-specific cellular immune responses [28][29][30]. A DNA vaccine harbors DNA-encoded antigens that are subjected to UPS degradation, resulting in peptide fragments that can be presented by MHC class I antigens to CD8+ T cells, mimicking a microbial infection. Although a DNA vaccine can break immune tolerance, it is unable to completely eliminate the viral particle load. In order to improve the efficacy of DNA vaccines, some strategies have been explored [31][32]. In this study, we aimed to develop a more powerful HBV DNA vaccine by constructing a plasmid that contains both the Ub and HBcAg genes.

Ubiquitin is a small, highly conserved regulatory protein that is ubiquitous in eukaryotes. The most significant function of ubiquitin in proteolysis is its role as a signal for a target protein to be recognized and degraded in the proteasome [33]. T cell receptors recognize short peptides that are presented by MHC class I molecules on antigen-presenting cells [34][35]. Higher rates of intracellular antigen traffic should increase the number and variety of peptides that are available for MHC class I binding, increasing the immune response to the expressed antigen. A very effective way of improving antigenic presentation by DNA vaccination is to fuse the Ub gene to the target antigen. A direct correlation between rapid ubiquitin-mediated processing of antigens and enhanced cell-mediated immune responses has been established [36]. It has also become apparent that ubiquitylation is a reversible reaction, in which ubiquitin chains are conjugated and deconjugated by ubiquitylating and deubiquitylating enzymes, respectively [37]. To prevent cleavage of the fusion gene by deubiquitination enzymes, we constructed an expression vector encoding HBcAg fused to Ub, in which the C-terminal glycine of Ub was replaced with alanine. In addition, the N-terminal Met residue of HBcAg was replaced by Arg. Thus, the fusion protein could be quickly recognized by the UPS, enhancing HBcAg degradation.

Previous attempts of tagging antigens with ubiquitin have placed Arg at the N-terminus to target them for rapid processing [38][39][40]. By RT-PCR, the cassettes of the Ub-HBcAg constructs were efficiently transcribed, and by western blot, HBcAg was translated from the expression vectors. As expected, ubiquitin-fused HBcAg converted into an excellent substrate for the UPS. We found that COS-7 cells that transfected with pUb-HBcAg expressed low levels of protein.

The function of our DNA vaccines was compared by measuring their effects on immune responses in BALB/c mice. We found that pUb-HBcAg enhanced the recruitment and activation of T cells and increased the number of antigen-specific CD8+/IFN-γ+ T cells. In this study, we noted enhanced lymphocyte proliferation in mice that were vaccinated with ubiquitinated HBcAg compared with nonubiquitinated HBcAg. This result shows that ubiquitin conjugation increased the cell-mediated immune response.

Antigen-specific CD8+ T cells play a vital role in the control of viral infections. They can remove infected target cells through cytotoxic or noncytotoxic activities, such as the production of IFN-γ and other Th1 cytokines [41]. It is believed that Th1 cells primarily secrete IL-2 and IFN-γ, whereas Th2 cells secrete the type II cytokines IL-4 and IL-10. We measured IL-2, IFN-γ, IL-10, and IL-4 in the spleens of mice that were immunized with our vaccines. Mice that were given ubiquitinated HBcAg expressed higher levels of IL-2 and IFN-γ compared versus nonubiquitinated HBcAg. T cell-derived cytokines can enhance antigen-specific T cell populations. In mice that were immunized by pUb-HBcAg, the number of HBcAg-specific CD8+/IFN-γ+ T cells in spleen was higher than in pHBcAg recipients, indicating that the immune responses are type 1 rather than type 2. The Th1 cell has been reported to correlate with the induction of CTL activity, which is beneficial for viral or tumor eradication [42][43]. Inadequate endogenous antigen presentation by MHC class I molecules to CD8+ T cells is one of the reasons why our immune system fails to eliminate pathogens. Patients with CHB or therapeutic failure present with deficient Th1 immunity, associated with inefficient CD8+ T cell cytotoxicity. In our study, the enhancement of antigen presentation increased the number of antigen-specific CD8+/IFN-γ+T cells in pUb-HBcAg-immunized mice. Ubiquitin-fused HBcAg is rapidly degraded by the proteasome, resulting in efficient production of a variety of peptides, including many CTL epitopes, that might be presented by many types of MHC class I molecules.

As shown in Figure 7, mice that were immunized with pUb-HBcAg 100 μg showed substantial cytotoxic activity against target cells, while those immunized with pUb-HBcAg 50 μg or pHBcAg showed remarkably enhanced cytotoxicity. We have clearly shown that immunization a ubiquitin-fuse HBcAg DNA vaccine induces robust HBcAg-specific immune responses in BALB/c mice, consistent with previous reports [44][45]. pUb-HBcAg can not only induce cell-mediated immune responses but also enhance humoral immune responses. We observed very high levels of anti-HBc IgG in mice that were immunized with pUb-HBcAg 100 μg compared with other plasmids. These results suggest that pUb-HBcAg elicits strong, specific anti-HBc humoral responses in BALB/c mice after DNA vaccination. However, in Vidalin's study [46], when ubiquitin was fused to hepatitis C virus core antigen, the antibody response was undetectable compared with the nonfusion vaccine. This difference in result might be attributed to the disparate antigenicities of the proteins.

Both pUb-HBcAg and pHBcAg induced humoral and cellular immune responses in normal mice, but pUb-HBcAg provoked a more robust response, suggesting that when the Ub gene is fused to the HBcAg gene, the efficacy of the DNA vaccine increases. Therefore, this novel strategy may have therapeutic value in infectious diseases and cancer.
